# Plexin Domain Containing 2, a Protein Specifically Expressed and Elevated in Human Pancreatic Cancer Tissue and Serum, Influences Cell Proliferation by Correlating With Cortactin

**DOI:** 10.1002/cam4.71459

**Published:** 2025-12-09

**Authors:** Junya Tsuboi, Akiko Eguchi, Hiroyuki Inoue, Masako Ichishi, Masatomo Go, Ryo Okajima, Noriko Yasuhara, Ryo Nakagawa, Mina Tempaku, Kiyora Izuoka, Takamitsu Tanaka, Kenji Nose, Naohiko Yoshizawa, Yoshifumi Hirokawa, Reiko Yamada, Kyosuke Tanaka, Takeshi Kawamura, Tetsuji Yamaguchi, Yoshiyuki Takei, Motoh Iwasa, Takefumi Yamashita, Masatoshi Watanabe, Hayato Nakagawa

**Affiliations:** ^1^ Department of Gastroenterology and Hepatology Mie University Graduate School of Medicine Tsu Japan; ^2^ Biobank Center Mie University Hospital Tsu Japan; ^3^ Department of Gastroenterology and Hepatology Goryokaku Hospital Hakodate Japan; ^4^ Department of Oncologic Pathology Mie University Graduate School of Medicine Tsu Japan; ^5^ Department of Physical Chemistry School of Pharmacy and Pharmaceutical Sciences, Hoshi University Shinagawa Japan; ^6^ Laboratory for Systems Biology and Medicine Research Center for Advanced Science and Technology, The University of Tokyo Meguro Japan; ^7^ Department of Life Science College of Humanities and Science Nihon University Chiyoda Japan; ^8^ Omiya City Clinic Saitama Japan; ^9^ Isotope Science Center The University of Tokyo Bunkyō Japan; ^10^ Department of Health Promotion and Preventive Medicine Mie University Graduate School of Medicine Tsu Japan

**Keywords:** AlphaFold3, biomarker, cortactin, pancreatic adenocarcinoma, plexin domain containing 2, therapeutic target

## Abstract

**Background:**

Pancreatic ductal adenocarcinoma (PDAC) is a highly aggressive cancer with limited treatment options. Plexin domain containing 2 (PLXDC2), a tumor endothelial marker, has been implicated in several cancers, but its role in PDAC remains unclear. This study aimed to investigate the role of PLXDC2 and its interaction with cortactin in PDAC.

**Method:**

PLXDC2 and cortactin expression levels were assessed by immunohistochemistry in human PDAC tissues. Serum PLXDC2 levels were measured using ELISA. PDAC cell lines were transfected with PLXDC2 siRNA. The effects of PLXDC2 knockdown on cortactin and oncogene expression, as well as cell proliferation, were evaluated using quantitative PCR, Western blotting, immunofluorescence, and proliferation assays. Computational modeling with AlphaFold3 predicted the 3D structure of the PLXDC2‐cortactin complex and designed inhibitory peptide.

**Results:**

PLXDC2 was overexpressed in PDAC tissues and serum, with an AUROC of 0.987 compared to healthy individuals. PLXDC2 co‐expressed with cortactin in human PDAC tissues. In PDAC cells, PLXDC2 knockdown led to decreased cortactin expression, followed by a reduction of oncogene (*c‐myc* and *Oct4)* expression and proliferation. Computational analysis predicted that the SH3 domain of cortactin binds to the PSI domain of PLXDC2. Peptide‐mediated inhibition of the PLXDC2‐cortactin binding, by targeting the SH3 domain of cortactin, led to a reduction of oncogenic and proliferative genes.

**Conclusions:**

PLXDC2 is a potential biomarker of PDAC diagnosis. Targeting the PLXDC2–cortactin interaction may offer a novel therapeutic strategy.

## Graphical abstract

In the predicted complex structure, PLXDC2 has a concave region where the SH3 domain of cortactin fits, forming a complex. In particular, we found that LYDYQ (500–504) of cortactin is included in the binding interface.

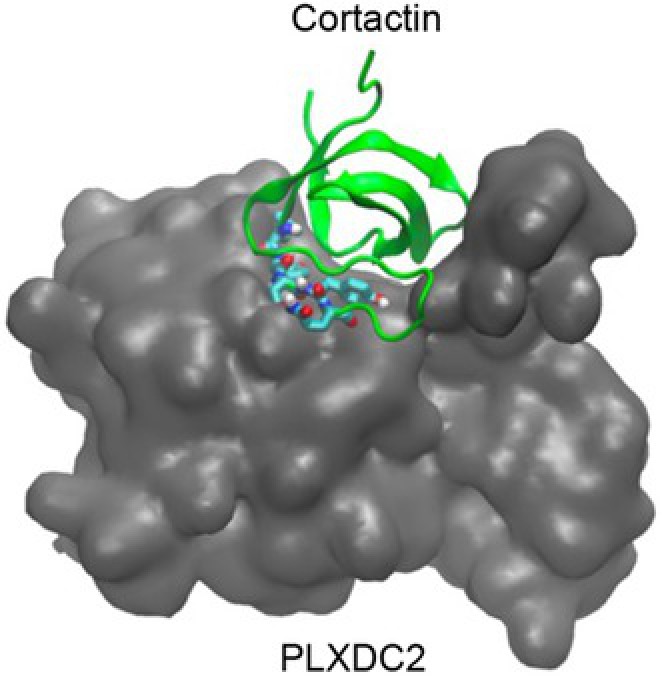

## 1 Introduction

Pancreatic cancer is one of the most lethal neoplasms, with a 5‐year relative survival rate of 13%. The incidence of pancreatic cancer continues to increase [1]. Pancreatic ductal adenocarcinoma (PDAC) is the most common type of pancreatic cancer, and most patients with PDAC have surgically inoperable disease due to its easy metastasis and delayed diagnosis. This is despite surgical excision being the only potentially curative treatment for PDAC [2]. Therefore, the diagnosis of PDAC at an early stage is critical to improve the prognosis of patients. Endoscopic ultrasound‐guided tissue acquisition (EUS‐TA) is the gold standard for definitive PDAC diagnosis [3]. While imaging modalities like ultrasound, MRI, and computed tomography (CT) are used for screening, non‐invasive biomarkers are preferred to minimize patient risk from invasive procedures and radiation exposure. Indeed, multiple factors including proteins, DNA and/or RNA have been identified as biomarkers for early detection and an improvement in prognosis [4]. Blood‐based liquid biological tests measuring serum CA19‐9 and elastase 1 levels are popular clinical tests. However, these levels are not always elevated in PDAC patients due to low accuracy, sensitivity, and specificity [5]. Moreover, these markers are elevated in other forms of cancer [6]. While various prognostic biomarkers, including cortactin, have been identified by immunohistochemistry (IHC) [7, 8], none of them has been clinically validated due to their inability to be detected in blood [9, 10]. KRAS mutations, which are present in 95% of PDAC patients, contribute to PDAC progression through the activation of several pathways, including MYC [11], and the abnormality of glucose metabolism [12]. Additionally, Oct4 is involved in the PDAC cell proliferation and invasion through the AKT pathway [13, 14], as part of the various molecular pathways identified for PDAC [15]. Currently, several clinical trials targeting KRAS mutations are underway globally [16], but novel therapeutic targets in PDAC are still needed.

Tumor endothelial markers (TEMs) were originally identified as endothelial cell surface markers linked to tumor‐specific angiogenesis and are specifically expressed in various malignant tumors [17, 18, 19, 20, 21, 22]. TEMs are released into the blood after being cleaved by the enzymes and are predicted to be associated with survival related to the progression of colon cancer [23, 24]. Plexin domain containing 2 (PLXDC2), also known as tumor endothelial marker 7‐related (TEM7R), has been identified as a member of the TEM family. However, current evidence indicates that PLXDC2 is expressed on various cell types [25, 26, 27]. Furthermore, PLXDC2 is associated with diverse malignant tumor behaviors [21, 28, 29], including those in human ovarian cancer [29], hepatocellular carcinoma (HCC) [30], colorectal cancer [31] and gastric cancer [32]. However, the role of PLXDC2 in pancreatic cancer has not been examined. PLXDC2 also interacts with cortactin, a protein that binds to PLXDC2's extracellular domain [33]. Cortactin, a family of actin‐binding proteins, is also known to promote invasive protrusions and degrade extracellular matrix in cancer cells, which is associated with tumor metastasis and poor prognosis [34]. In this study, we investigate the role of PLXDC2 and its interaction with cortactin in human PDAC. We also explore the potential of serum PLXDC2 levels as a biomarker in PDAC patients.

## 2 Material and Methods

### 2.1 Patients and Samples

The study protocol was approved by the ethics committee of Mie University Hospital and Omiya City Clinic (H2023‐090). This study was performed retrospectively on stored samples, and subjects were allowed to opt out of their data being used. Written informed consent was obtained from all subjects at the time of blood sampling. The research project was performed in accordance with the ethical standards of the Declaration of Helsinki. All patients with PDAC were diagnosed cytologically or histologically and were operated on between August 2018 and March 2020 at Mie University Hospital. Patient clinical data, including age, gender, tumor size, tumor location, TNM stage, tumor differentiation, and the presence or absence of neoadjuvant chemoradiotherapy, were collected. The histology of PDAC tissue was assessed according to the criteria of the 2019 WHO classification of pancreatic tumors [35]. The pathological TNM status was assessed according to the criteria of the 8th edition of TNM classification of the Union for International Cancer Control. Serum samples were kept at −80°C until PLXDC2 measurement using a human PLXDC2 ELISA (My BioSource, San Diego, CA, USA) according to the manufacturer's instructions.

### 2.2 Immunohistochemistry of PLXDC2 and Cortactin Expressions

Immunohistochemistry for PLXDC2 was performed as described previously [30, 31]. Briefly, paraffin‐embedded tissue samples were sectioned at a thickness of 5 μm and stained with an anti‐PLXDC2 rabbit monoclonal antibody #4G3 (Abwiz Bio, San Diego, CA, USA) or an anti‐cortactin antibody (Santa Cruz Biotechnology, Dallas, TX, USA), followed by peroxidase‐conjugated secondary antibody (Vector) according to the manufacturer's instructions. Images were taken using a microscope (Olympus DP22 and DP2‐SAL, Japan).

### 2.3 In Vitro Cell Culture Studies

Panc‐1 and KP‐3 cells were grown and maintained in RPMI1640 (Thermo Fisher Scientific, Waltham, MA, USA) supplemented with 10% fetal bovine serum (Cytiva, USA), penicillin, and streptomycin (Gibco) at 37°C in a 5% CO2 incubator. Panc‐1 and KP‐3 cells were treated with 15 nM of either PLXDC2 or negative control siRNA (OriGene, Rockville, MD, USA) using RNAiMax transfection reagent (Thermo Fisher). Cells were harvested at 24 h post‐treatment for RNA extraction or at 72 or 48 h post‐treatment for protein extraction in Panc‐1 or KP‐3, respectively. In addition, cell proliferation was quantified using a Cell‐counting kit‐8 (DOUJIN, Japan) at 48 h post‐treatment. Panc‐1 cells were treated with 40 μM of the peptide, NH_2_‐TAVALYDYQ‐Palmitoyl (Eurofins Japan, JAPAN), and harvested at 24 h post‐treatment for RNA extraction.

### 2.4 Real‐Time PCR

Total RNA was isolated from cells using TRI Reagent (Molecular Research Center, Cincinnati, OH, USA) according to the manufacturer's instructions. cDNA was synthesized from the total RNA using a cDNA Synthesis kit (Takara Bio, Kusatsu, Japan). Real‐time PCR quantification was performed using the SYBR Green PCR mixture (Thermo Fisher Scientific Inc) and the QuantStudio 1 (Thermo Fisher Scientific Inc). The PCR primers used to amplify each gene are listed in Table S1. Mean values of mRNA were normalized to beta 2 microglobulin (β2m).

### 2.5 Immunoblot Analysis

For Western blot analysis, 20 μg of whole‐cell lysate was resolved by TGX precast gels and transferred to a nitrocellulose membrane (BioRad, Hercules, CA, USA). The blotted membranes were incubated with an anti‐PLXDC2 antibody (Abwiz Bio) and an anti‐cortactin antibody (Santa Cruz Biotechnology), followed by a peroxidase‐conjugated secondary antibody (GE Healthcare Life Sciences, Pittsburgh, PA, USA). Protein bands were visualized using enhanced chemiluminescence reagents (Thermo Fisher Scientific) and digitized using a CCD camera (LAS4000 mini; Fujifilm, Japan). Expression intensity was quantified by Multi Gauge (Fuji). Protein loading was verified using an anti‐β‐actin (BioVision, CA, USA) antibody.

### 2.6 Computational Prediction of the Protein Complex

The AI‐based complex structure prediction was performed using AlphaFold3 [36]. The input protein sequences were obtained from UniProt [the entry IDs for PLXDC2 and Cortactin are Q6UX71 and Q14247, respectively]. First, the structure prediction of Cortactin revealed that only the SH3 domain (residues 492–550) at the C‐terminal region forms a rigid three‐dimensional structure. This SH3 domain has also been suggested in previous studies [25] to interact with PLXDC2. Using the sequence of the Cortactin SH3 domain and the full‐length sequence of PLXDC2 as inputs for AlphaFold3, we calculated the complex structure of Cortactin and PLXDC2. It was found that Cortactin interacts with the rigid domain of PLXDC2 (residues 107–372), which includes the PSI domain (residues 327–372). However, since the accuracy of the binding structure prediction was not sufficiently high, we re‐calculated the complex structure by excluding all disordered regions and focusing solely on the rigid regions. The input regions were the SH3 domain of Cortactin (residues 492–550) and the rigid domain of PLXDC2 (residues 107–372).

Additionally, we performed complex structure prediction using the docking method. The calculations were conducted using the ZDOCK program [37] and the HADDOCK program [38]. For these calculations, we used the SH3 domain structure of Cortactin (residues 492–550) and the rigid domain structure of PLXDC2 (residues 107–372) predicted by AlphaFold3. First, rigid docking was performed using ZDOCK to explore the global complex structure. To refine the highest‐scoring structure from the ZDOCK calculation, we conducted flexible docking using HADDOCK. Here, PLXDC2 residues (residues 186–196 and 296–302) and the Cortactin SH3 domain were interface residues in the HADDOCK calculation, based on the ZDOCK results.

### 2.7 Statistical Analysis

All data are expressed as the mean ± SEM and median (interquartile range [IQR]). Data were analyzed using a t‐test or Mann–Whitney *U* test for two groups. The optimal cutoff value that maximized the sum of sensitivity and specificity was selected using receiver operating characteristic (ROC) curve analysis for the identification of PDAC. All statistical analyses were performed using Prism (GraphPad Software Inc., CA, USA). Differences were considered significant at *p* < 0.05.

## 3 Results

### 3.1 PLXDC2 Is Expressed in Human PDAC Tissues

The study group included six surgically resected specimens from six patients with PDAC (three males and three females). The characteristics of these six PDAC patients were shown in Table 1. The mean age of the patients was 64 ± 3.5 years (range: 57–80). The mean tumor size was 25 ± 5.0 mm (range: 17–50). The tumor was located in the pancreatic head in all patients. The cohort consisted of patients with earlier TNM stages: three with stage IA and three with stage IIB. Tumor differentiation was found to be well differentiated in three patients and poor to moderate in three patients within our cohort. Three patients received preoperative chemoradiotherapy, and the remaining three patients underwent immediate surgery.

**TABLE 1:** Demographic and clinical characteristics of PDAC tissues.

Case no.	Gender	Age (years)	Tumor size (mm)	Tumor location	T	N	M	Stage	Tumor differentiation	Neoadjuvant chemoradiotherapy
A	Female	58	50	Head	T3	N1	M0	IIB	Poor‐modulate	+
B	Male	64	20	Head	T1	N0	M0	IA	Modulate	−
C	Male	57	22	Head	T2	N1	M0	IIB	Poor	−
D	Female	61	26	Head	T2	N1	M0	IIB	Well	+
E	Male	68	20	Head	T1	N0	M0	IA	Well	+
F	Female	80	17	Head	T1	N0	M0	IA	Well	−

**FIGURE 1:**
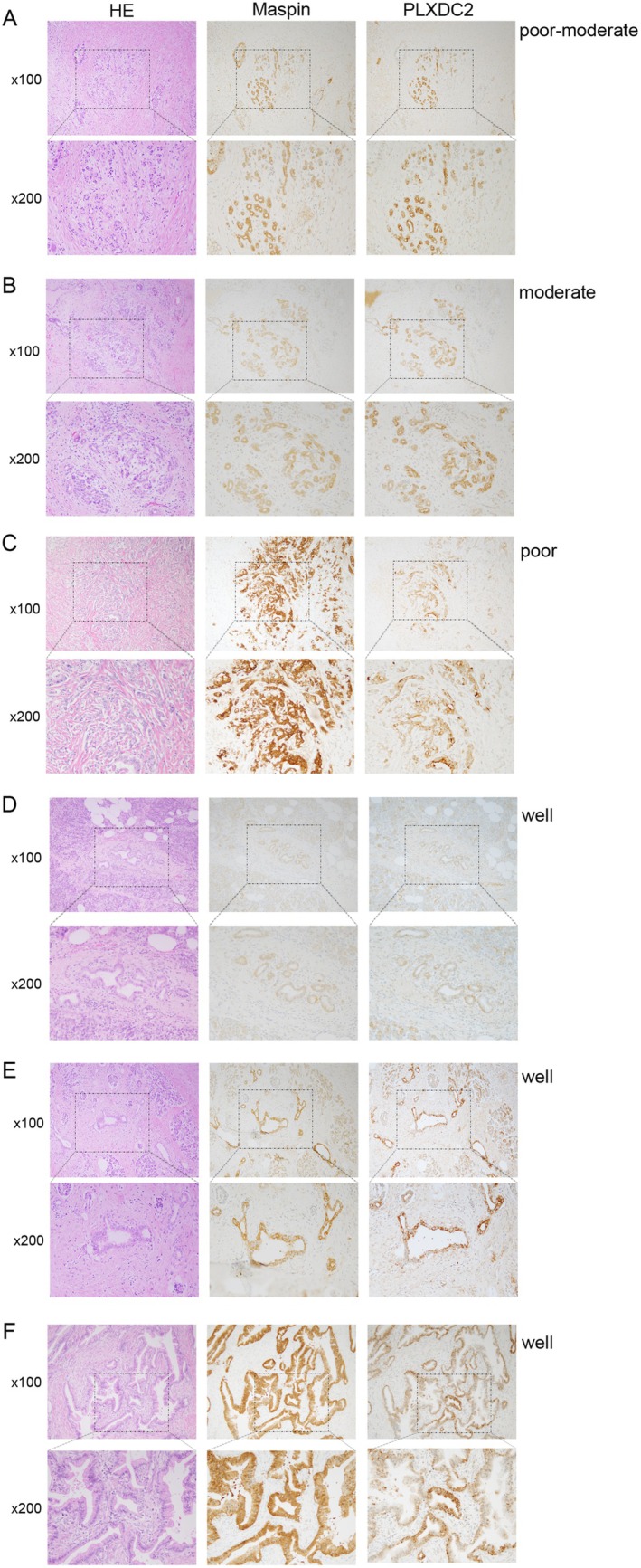
PLXDC2 expression in human PDAC tissues. (A–F) Pathological images of six human PDAC tissues. The images of HE, Maspin staining as a PDAC marker and PLXDC2 stainings ×100 and ×200 of sections from PDAC samples. HE, hematoxylin and eosin; PDAC, pancreatic ductal adenocarcinoma; PLXDC2, plexin domain containing 2.

We present the immunohistochemical staining of PLXDC2 expression in human PDAC tissues from six different patients (Table 1). Figure 1 shows representative stained images of hematoxylin and eosin (HE), maspin to identify specific PDAC, and PLXDC2. The images are shown at 100× magnification for the upper images and 200× for the lower images (Figure 1A–F). PLXDC2 is expressed in the PDAC area, as shown by maspin positivity (Figure 1A–F). PLXDC2 is strongly expressed in PDAC tissues with poor‐to‐moderate tumor differentiation (Figure 1A–C), showing a similar expression pattern to maspin staining. In contrast, PLXDC2 expression is weak or patchy (a mixture of weak and strong) in PDAC tissues with well‐differentiated tumors (Figure 1D–F). PLXDC2 expression is not altered by the presence of chemoradiotherapy (Figure 1A,D,E) compared to its absence (Figure 1B,C,F), indicating that chemoradiotherapy does not affect PLXDC2 expression in PDAC. These results suggest that PLXDC2 is expressed in human PDAC tissue, and its expression is not affected by chemoradiotherapy.

### 3.2 Serum PLXDC2 Levels Are Significantly Elevated in PDAC Patients

**FIGURE 2:**
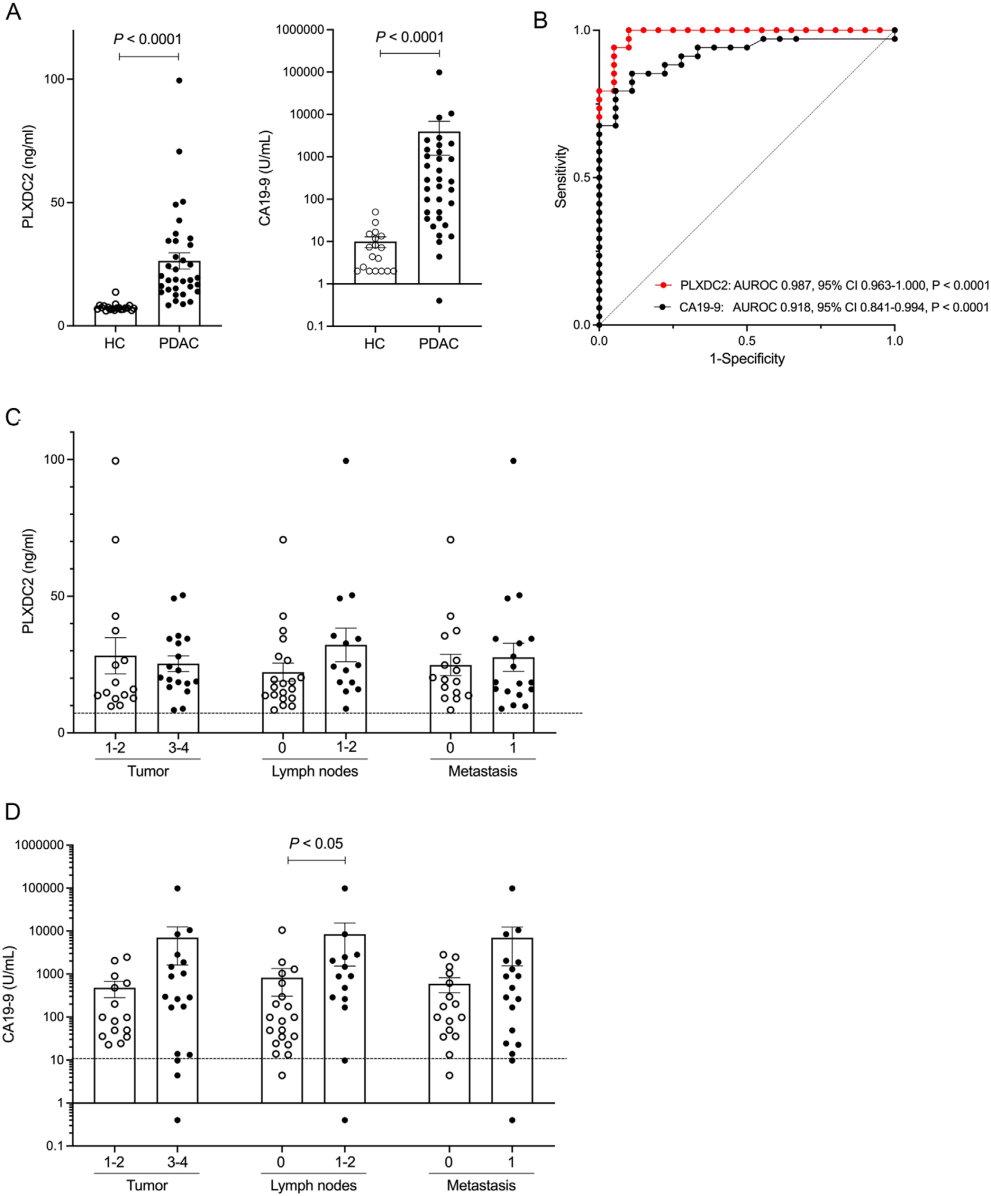
Serum PLXDC2 levels in patients with PDAC. (A) Serum PLXDC2 and CA19‐9 levels between PDAC patients and healthy individuals. (B) ROC curve of serum PLXDC2 and CA19‐9 levels between PDAC patients and healthy individuals. (C, D) Serum PLXDC2 (C) or CA19‐9 (D) levels in TNM classification of PDAC patients. Dotted lines indicate the cut‐off value of PLXDC2 (C) and the standard value of CA19‐9 (D). HC, healthy controls; PDAC, pancreatic ductal adenocarcinoma; PLXDC2, plexin domain containing 2.

The expression of PLXD2 in PDAC tissue and its characterization, which is cleaved from the plasma membrane by enzymes [25], led us to further investigate whether PLXDC2 levels are elevated in the blood. Serum PLXDC2 levels were measured in 34 PDAC patients and 20 healthy individuals. There were statistically significant differences in age (median: 74.0 for PDAC patients vs. 53.5 for healthy individuals) and sex (male: 44.1% for PDAC patients vs. 75.0% for healthy individuals), with *p* < 0.001 and *p* < 0.05, respectively (Table 2). Tumor location, tumor size, TNM classification, and TNM stage in PDAC patients were described in Table 2. Serum PLXDC2 levels were significantly elevated in patients with PDAC (mean: 26.3 ng/mL) compared to healthy individuals (mean: 7.52 ng/mL) (*p* < 0.01) (Figure 2A). Serum CA19‐9 levels were also significantly elevated in PDAC patients (mean: 3976 U/mL) compared to healthy individuals (mean: 10.05 U/mL) (Figure 2A). ROC analyses yielded AUC values of 0.987 (95% CI: 0.963–1.000; *p* < 0.0001) and 0.918 (95% CI: 0.841–0.994; *p* < 0.0001) for serum PLXDC2 and CA19‐9 levels, respectively (Figure 2B). We calculated the cutoff value of serum PLXDC2 levels to be 8.3 ng/mL with a sensitivity of 100% and a specificity of 90%. Notably, none of the 34 (0%) PDAC patients had serum PLXDC2 levels below the cutoff value (8.3 ng/mL), whereas nine out of 34 (26.5%) PDAC patients had serum CA19‐9 levels (0.4 to 35.4 ng/mL) below the standard value (37 ng/mL). This suggests that serum PLXDC2 may be a better marker for identifying PDAC patients compared to CA19‐9. In the tumor (T), lymph node (N), and metastasis to distant organs (M) classification, serum PLXDC2 levels tended to increase in the presence of lymph node metastasis (N1‐2) (mean: 32.19 ng/mL) compared to the absence of lymph node metastasis (N0) (mean: 22.24 ng/mL) (*p* = 0.09) (Figure 2C). Serum CA19‐9 levels were increased in T3‐4 (mean: 7039 U/mL) compared to T1‐2 (mean: 479.9 U/mL), in N1‐2 (mean: 8476 U/mL) compared to N0 (mean: 826.4 U/mL) (*p* < 0.05), and in M1 (mean: 6983 U/mL) compared to M0 (mean: 594.1 U/mL) (Figure 2D). These results reveal that serum PLXDC2 levels can be used to distinguish PDAC from healthy individuals.

**TABLE 2:** Characteristics of PDAC patients and healthy individuals.

	PDAC patients (*n* = 34)	Healthy individuals (*n* = 20)	*p*
Age, median (IQR)	74.0 (68.3–80.3)	53.5 (46.8–59.5)	< 0.001
Sex, male, no. (%)	15 (44.1)	15 (75.0)	0.046
PLXDC2 (ng/mL), median (IQR)	19.1 (14.8–34.0)	7.1 (6.7–7.7)	< 0.0001
CA19‐9 (U/mL), median (IQR)	231.9 (38.8–1227.8)	5.8 (2.0–13.1)	< 0.0001
Tumor location, no. (%)	Head: 20 (58.8)		
	Body: 7 (20.6)		
	Tail: 7 (20.6)		
Tumor size (mm), median (IQR)	34 (23–43)		
T stage, no. (%)	Tis: 1 (2.9)		
	T1: 7 (20.6)		
	T2: 8 (23.5)		
	T3: 5 (14.7)		
	T4: 13 (38.2)		
N stage, no (%)	N0: 20 (58.8)		
	N1: 8 (23.5)		
	N2: 6 (17.6)		
M stage, no (%)	M0: 16 (47.1)		
	M1: 18 (52.9)		
Stage (8th edition), no (%)	IA: 6 (17.6)		
	IB: 2 (5.9)		
	IIA: 2 (5.9)		
	IIB: 1 (2.9)		
	III: 5 (14.7)		
	IV: 18 (52.9)		

*Note:* Stage classification follows the 8th edition of the UICC TNM system.

Abbreviations: IQR, interquartile range; PDAC, pancreatic invasive ducatal adenocarcinoma.

### 3.3 PLXDC2 Is Co‐Localized With Cortactin in PDAC Patients

**FIGURE 3:**
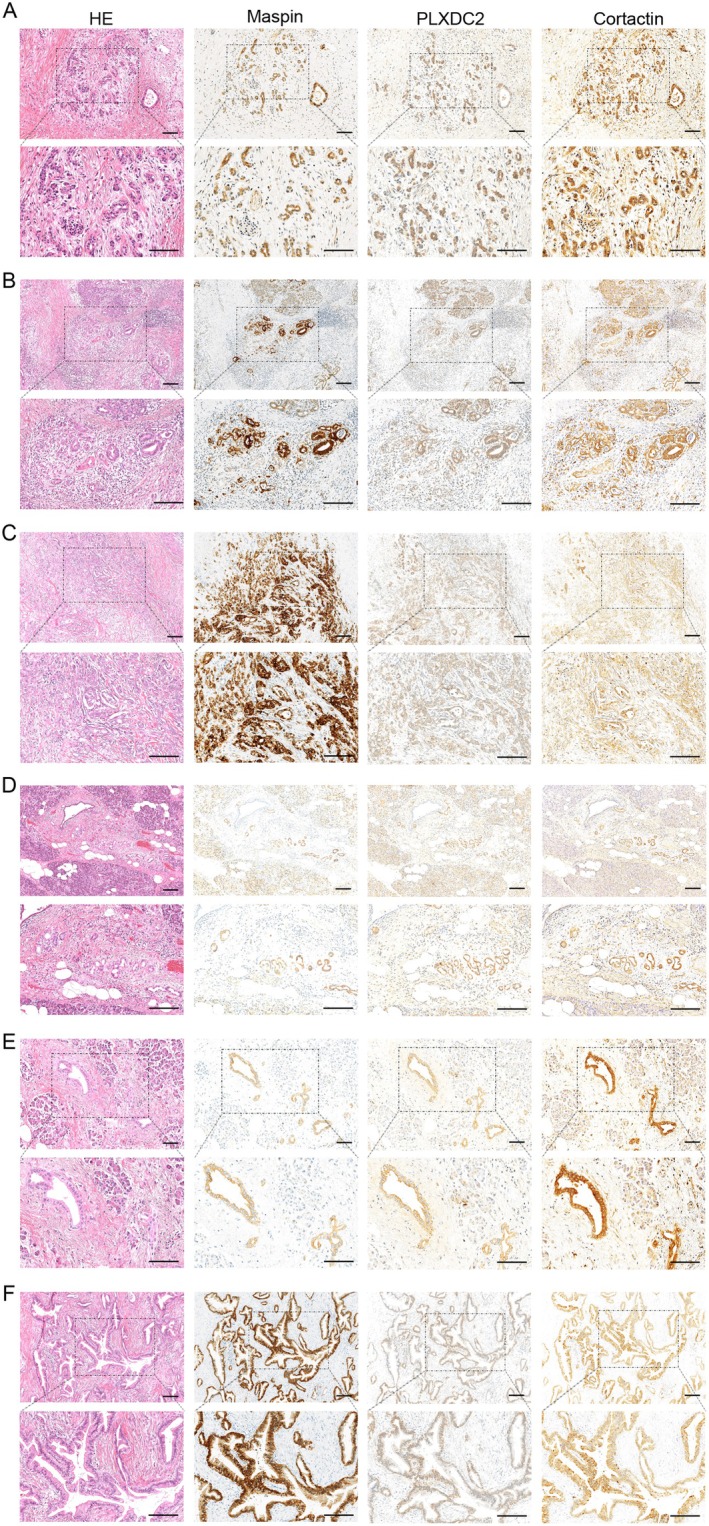
PLXDC2 and cortactin expressions in human PDAC tissues. (A–F) Pathological images of four human PDAC tissues. The images of HE, Maspin staining as a PDAC marker, plexin domain containing protein 2 (PLXDC2) and cortactin stainings of sections from PDAC samples. Scale bar, 100 μm. HE, hematoxylin and eosin; HE, hematoxylin and eosin; PDAC, pancreatic ductal adenocarcinoma; PLXDC2, plexin domain containing 2.

To investigate the role of PLXDC2 in PDAC, we detected the co‐localization of PLXDC2 with cortactin. Cortactin has been reported to be expressed in human PDAC tissue [39] and has been identified as a protein capable of binding to the extracellular region of PLXDC2. We present the immunohistochemical staining of PLXDC2 and cortactin expressions in human PDAC tissues from six different patients (Figure 3; Table 1). The images were shown at low magnification for the upper images and high magnification for the lower images (Figure 3A–F).

Notably, PLXDC2 was co‐localized with cortactin, suggesting that PLXDC2 might be involved in the formation of invasive protrusions together with cortactin, as previously reported in gastric cancer.

### 3.4 The Correlation Between PLXDC2 and Cortactin Influences the Expression of Oncogenes, c‐Myc and Oct4, and Cell Proliferation in Human PDAC Cell Lines

**FIGURE 4:**
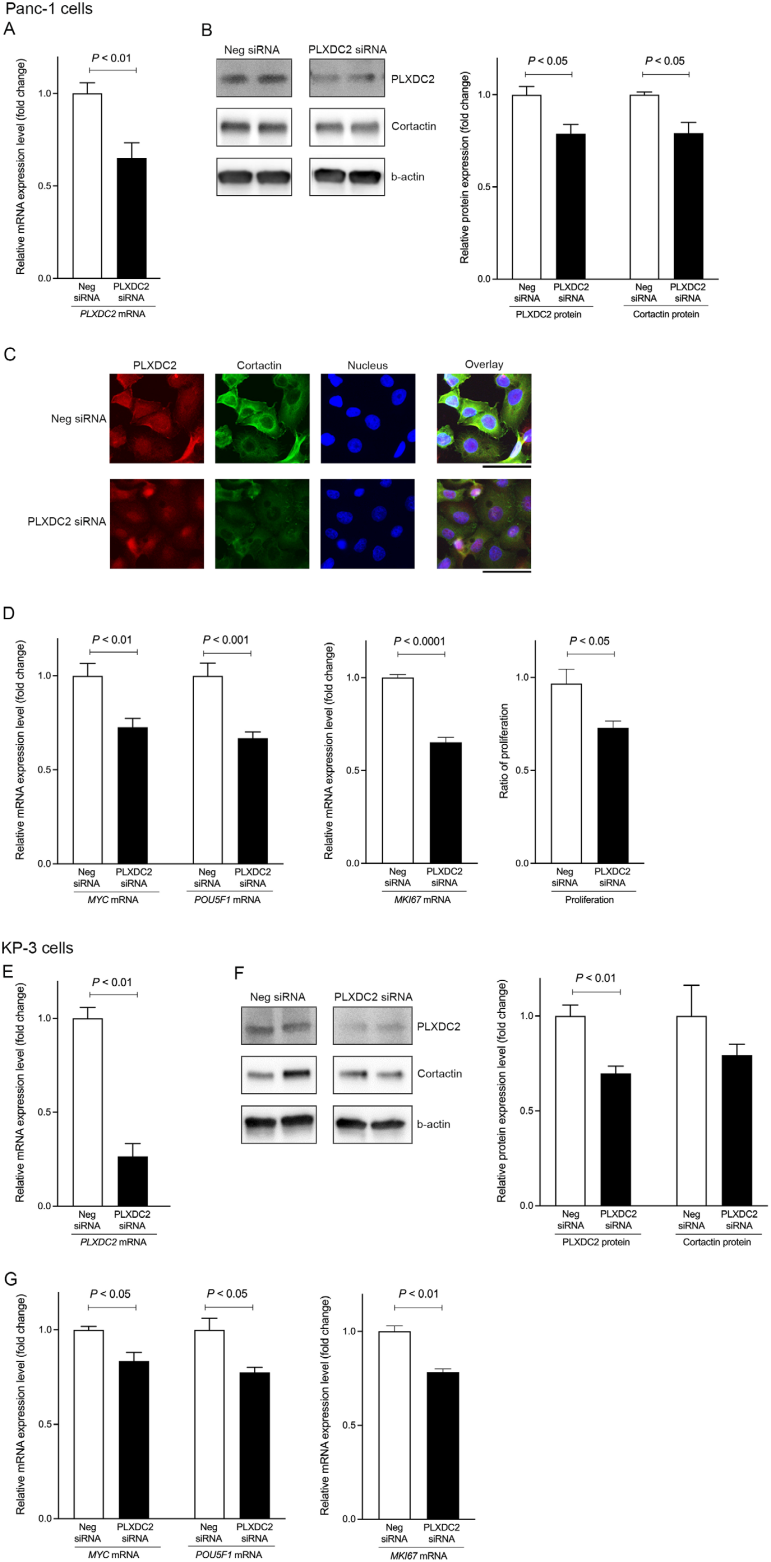
Correlation between PLXDC2 and cortactin in human PDAC cells. (A) Gene expression of *PLXDC2* in Panc‐1 cells treated with Neg siRNA as a control and PLXDC2 siRNA. (B) Immunoblotting of PLXDC2, cortactin and b‐actin as a control in Panc‐1 cells treated with Neg siRNA and PLXDC2 siRNA. The quantification of PLXDC2 and cortactin expression from the immunoblotting. (C) Immunostaining for PLXDC2 (red), cortactin (green) and nucleus (blue) in Panc‐1 cells treated with Neg siRNA and PLXDC2 siRNA. Scale bar, 50 μm. (D) Gene expression of *MYC*, *POU5F1*, and *MKI67*, as well as proliferation, in Panc‐1 cells treated with Neg siRNA and PLXDC2 siRNA. (E) Gene expression of *PLXDC2* in KP‐3 cells treated with Neg siRNA as a control and PLXDC2 siRNA. (F) Immunoblotting of PLXDC2, cortactin and b‐actin as a control in KP‐3 cells treated with Neg siRNA and PLXDC2 siRNA. The quantification of PLXDC2 and cortactin expression from the immunoblotting. (G) Gene expression of *MYC*, *POU5F1*, and *MKI67* in KP‐3 cells treated with Neg siRNA and PLXDC2 siRNA. Abbreviations: PLXDC2, plexin domain containing 2; PDAC, pancreatic ductal adenocarcinoma; GAPDH, Glyceraldehyde‐3‐phosphate dehydrogenase; POU5F1, POU domain class 5 transcription factor 1; MKI67, marker of proliferation Ki‐67.

To explore the correlation between PLXDC2 and cortactin, we suppressed PLXDC2 using siRNAs and examined cortactin expression in a human PDAC cell line. *PLXDC2* mRNA levels (*p* < 0.01) were significantly suppressed in Panc‐1 cells treated with PLXDC2 siRNAs compared to those treated with negative control siRNAs (Figure 4A). In addition, PLXDC2 protein expression was significantly decreased in Panc‐1 cells, as assessed by immunostaining (*p* < 0.05) (Figure 4B; Figure S1) and immunofluorescence (Figure 4C). This led to a significant reduction of cortactin protein expression in PLXDC2‐suppressed cells compared to control cells, as assessed by immunostaining (*p* < 0.05) (Figure 4B; Figure S1) and immunofluorescence (Figure 4C). The simultaneous reduction of PLXDC2 and cortactin led to a significant decrease in *MYC* (*p* < 0.01) and *POU5F1* (*p* < 0.001) mRNA levels, which are crucial for PDAC cell proliferation (Figure 4D) [14, 40]. Indeed, *MKI67* mRNA levels, a proliferation gene, were significantly decreased in PLXDC2‐suppressed cells (*p* < 0.0001), resulting in a significant reduction of cell proliferation (*p* < 0.05) (Figure 4E). To confirm the results of the PLXDC2‐cortactin effects, we suppressed PLXDC2 using siRNAs in a different human PDAC cell line, KP‐3 cells. *PLXDC2* mRNA levels were significantly suppressed in KP‐3 cells treated with PLXDC2 siRNAs compared to negative control siRNAs as a control (*p* < 0.01) (Figure 4E). In addition, PLXDC2 protein expression was significantly decreased in KP‐3 cells, as assessed by immunostaining (*p* < 0.01) (Figure 4F; Figure S1), leading to a reduction of cortactin protein expression in PLXDC2‐suppressed cells compared to control cells (Figure 4F; Figure S1). The simultaneous reduction of PLXDC2 and cortactin led to a significant decrease in *MYC* (*p* < 0.05), *POU5F1* (*p* < 0.05), and *MKI67* (*p* < 0.01) mRNA levels (Figure 4G). These results suggest that the PLXDC2‐cortactin axis is part of the PDAC progression pathway.

### 3.5 The Binding Structure of PLXDC2 to Cortactin Was Computationally Revealed, and Its Structure Involved in PDAC Proliferation

**FIGURE 5:**
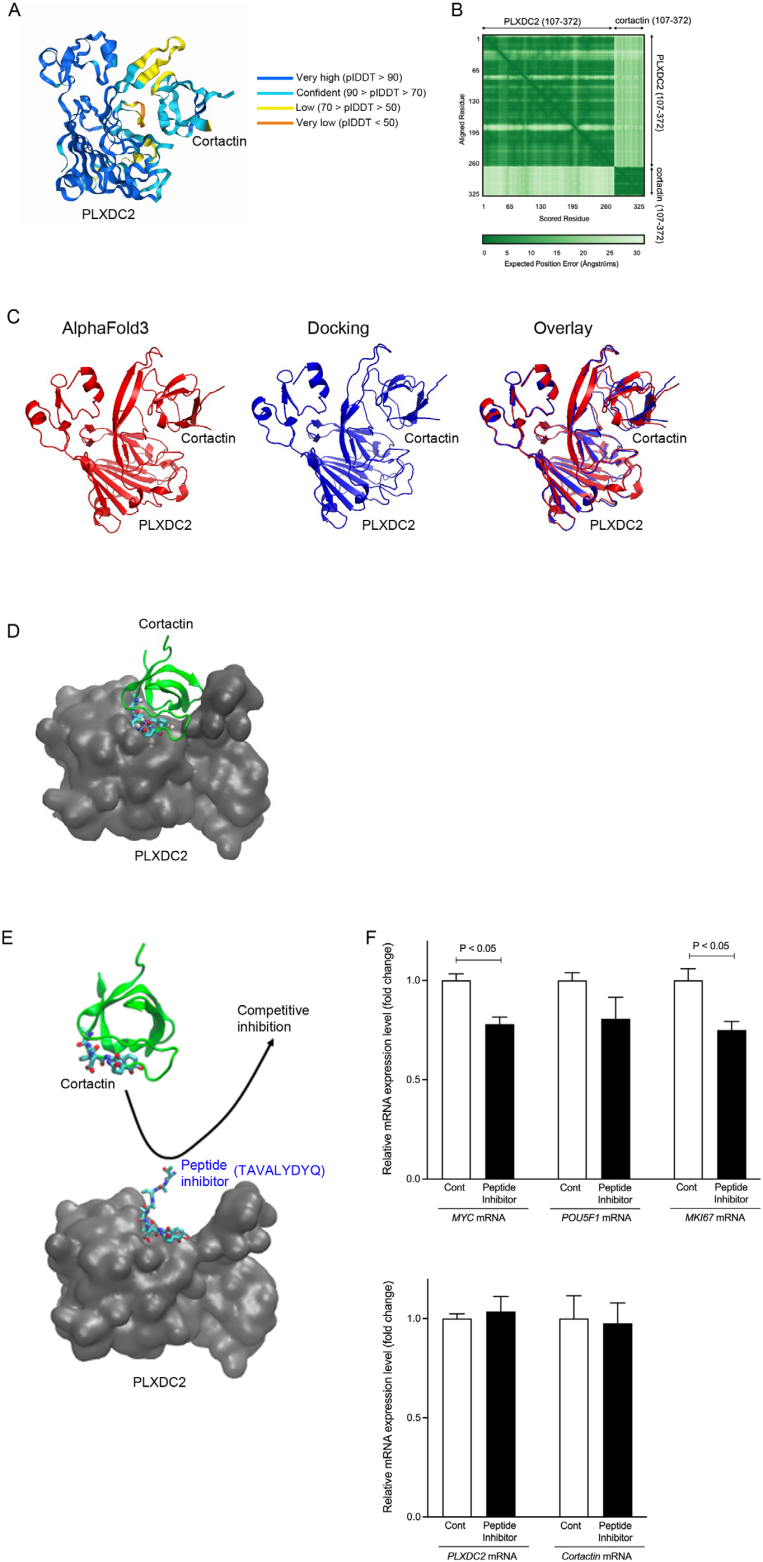
Computational structural analysis of protein–protein interaction between PLXDC2 and Cortactin. (A, B) pLDDT (A) and pAE (B) scores for the structured domains of PLXDC2 (107–372) and cortactin (492–550) using the AlphaFold3. (C) PLXDC2‐cortactin binding structure by AlphaFold3 (red) and Docking (blue) as a different method. (D) Predicted structure of PLXDC2‐cortactin complex. The SH3 domain of cortactin fits into a concave region of PLXDC2. (E) Predicted structure of PLXDC2 and competitive inhibitor. (F) Gene expression of *MYC*, *POU5F1*, *MKI67*, *PLXDC2*, and *cortactin* in Panc‐1 cells treated with peptide inhibitor. MKI67, marker of proliferation Ki‐67; PLXDC2, plexin domain containing 2; POU5F1, POU domain class 5 transcription factor 1.

To understand the physical basis of the interaction between PLXDC2 and cortactin, we predicted the binding structure of the structured domains of PLXDC2 (107–372) and cortactin (492–550) using the latest AI‐based software, AlphaFold3. Notably, these structured domains were identified based on the formation of secondary structures and high confidence scores (predicted local distance difference test, pLDDT) in the monomer structure predictions made by AlphaFold2. Although AlphaFold3 generated the PLXDC2‐cortactin binding structure (Figure 5A), the pLDDT was not very high (Figure 5A), and the predicted aligned error (pAE) score was not very low, as indicated by the light green color (Figure 5B). To confirm the reliability of the AlphaFold3 prediction from a different perspective, we conducted a physics‐based prediction (docking). The result of the docking prediction (Figure 5C, red) was similar to that of the AlphaFold3 prediction (Figure 5C, blue). Since the structure predictions calculated through the two independent methods were in close agreement (the ligand RMSD value was 3.5 Å) (Figure 5C, overlay), we consider the predicted structure to be highly reliable. In the predicted complex structure, PLXDC2 has a concave region where the SH3 domain of cortactin fits, forming a complex (Figure 5D). In particular, we found that LYDYQ (500–504) of cortactin is included in the binding interface, which was also identified as an important site for the binding of cortactin to PLXDC2 in a previous study [25]. These results show that the predicted structure of the PLXDC2‐cortactin complex suggests that the SH3 domain of cortactin binds to the PSI domain of PLXDC2. Based on this structural insight, we then explored the functional role of this interaction in tumor progression.

To disrupt the PLXDC2‐cortactin interaction, we synthesized the peptide NH_2_‐TAVALYDYQ‐Palmitoyl. This peptide was designed based on the LYDYQ (500–504) sequence of cortactin, which was identified as a critical binding site for PLXDC2 through both AlphaFold‐3 modeling and a previous study [25]. Palmitoylation enhances the hydrophobicity of the peptide, thereby promoting its membrane localization. Peptide‐mediated inhibition of the PLXDC2‐cortactin binding led to a reduction in the mRNA levels of the tumor‐related genes *MYC* (*p* < 0.05) and *POU5C1*, and the proliferation‐related gene *MKI67* (*p* < 0.05) (Figure 5E), while the mRNA levels of *PLXDC2* and *cortactin* remained unchanged (Figure 5E). These results suggest that targeting this protein–protein interaction could be a novel therapeutic strategy.

## 4 Discussion

We demonstrated that PLXDC2 is expressed in human PDAC tissues and is released into the blood, resulting in significantly elevated serum PLXDC2 levels compared to healthy individuals. Furthermore, we also showed that PLXDC2 was co‐localized with cortactin, which is involved in the formation of invasive protrusions, in human PDAC tissue. In an in vitro assay, suppression of PLXDC2 led to a reduction of cortactin followed by the down‐regulation of oncogenes and a decrease in cell proliferation.

We observed that PLXDC2 was expressed in the cytoplasm of PDAC, a pattern consistent with our previous reports on human HCC [30] and colorectal cancer [31]. In human colorectal cancer, PLXDC2 expression was increased in the invasive area [31], suggesting that PLXDC2 may also be associated with PDAC progression. Indeed, we found that PLXDC2 expression in PDAC tissues was co‐localized with cortactin, which has been identified as playing a role in invasive protrusion formation. As a note, we used a highly specific and low‐background anti‐PLXDC2 rabbit antibody (clone 4G3) [41], which allowed us to specifically detect PLXDC2 expression in the tumor area of PDAC, as well as in HCC and colorectal cancers in our previous studies [30, 31].

This is the first report indicating that TEMs, particularly PLXDC2, may be useful as a non‐invasive biomarker in human PDAC. The TEMs family, including PLXDC2, is known to be released into the blood after cleavage by cellular enzymes [42, 43]. Indeed, serum levels of TEMs such as TEM1 and TME8 have been reported to be associated with the TNM stage and to serve as a prognostic factor for the survival of patients with colorectal cancers [23, 24]. In this study, we showed that serum PLXDC2 levels were significantly elevated in patients with PDAC compared to healthy individuals, with an AUROC of 0.987. In contrast, the AUROC of CA19‐9 typically ranges from 0.85 to 0.9 [44], while it was 0.918 in this study, suggesting that serum PLXDC2 may have a higher diagnostic capability. The detection of PDAC using serum PLXDC2 levels offers several advantages. First, none of the PDAC patients in this study had serum PLXDC2 levels below the average found in healthy individuals. Second, serum PLXDC2 levels may reflect cortactin expression in human PDAC tissues due to the binding of PLXDC2 with cortactin. Although cortactin has been identified as a prognostic factor in PDAC tissues, it is not released into the blood.

We revealed that the association of PLXDC2 with cortactin is involved in the proliferation of PDAC cells, and its blockade led to a reduction in oncogene expression. Suppressing PLXDC2 led to a reduction in cortactin protein levels, followed by a decrease in key oncogenes, such as *MYC* and *POU5F1*, and a reduction in PDAC cell proliferation. The predicted structure of the PLXDC2‐cortactin complex suggests that the SH3 domain of cortactin, particularly the LYDYQ (500–504) motif (which was identified as a crucial binding site for PLXDC2) [25], binds to the PSI domain of PLXDC2. Previous studies have reported that cortactin is highly expressed in human PDAC tissues and contributes to the migration and invasion of PDAC cells [7]. Therefore, the PLXDC2‐cortactin complex may play a significant role in PDAC progression, although further research is needed to fully understand the role of this interaction.

This study has several limitations, including its retrospective design, small sample size, and an imbalanced population of PDAC patients and healthy individuals. Therefore, a large, multi‐center study with a more age‐ and sex‐balanced population is needed to validate these results. Furthermore, while the siRNA knockdown results are convincing, the lack of rescue experiments, such as re‐expressing PLXDC2, prevents full confirmation of specificity of the PLXDC2‐cortactin‐proliferation pathway. Future studies should include these experiments and also utilize primary PDAC cells to further validate our findings.

In conclusion, we are the first to report on the role of PLXDC2 in PDAC. We found that PLXDC2 is expressed in human PDAC tissue and is released into the bloodstream, resulting in elevated serum levels. This suggests the potential for using serum PLXDC2 as a non‐invasive biomarker for PDAC detection. Moreover, we demonstrated that PLXDC2 interacts with cortactin, which promotes the proliferation of PDAC cells. These findings suggest that PLXDC2 could be a potential therapeutic target for inhibiting PDAC proliferation.

## Author Contributions

**Junya Tsuboi:** data curation (lead), resources (lead), writing – original draft (lead). **Akiko Eguchi:** conceptualization (lead), formal analysis (lead), investigation (lead), writing – review and editing (lead). **Hiroyuki Inoue:** data curation (supporting), writing – review and editing (supporting). **Masako Ichishi:** data curation (equal), methodology (equal), writing – review and editing (supporting). **Masatomo Go:** data curation (equal), writing – review and editing (supporting). **Ryo Okajima:** data curation (supporting), writing – review and editing (supporting). **Ryo Nakagawa:** resources (equal), writing – review and editing (supporting). **Mina Tempaku:** data curation (equal), writing – review and editing (supporting). **Kiyora Izuoka:** data curation (equal), writing – review and editing (supporting). **Takamitsu Tanaka:** data curation (equal), writing – review and editing (supporting). **Kenji Nose:** data curation (equal), writing – review and editing (supporting). **Naohiko Yoshizawa:** data curation (equal), writing – review and editing (supporting). **Yoshifumi Hirokawa:** data curation (equal), writing – review and editing (supporting). **Reiko Yamada:** data curation (equal), writing – review and editing (supporting). **Kyosuke Tanaka:** data curation (equal), writing – review and editing (supporting). **Takeshi Kawamura:** data curation (equal), writing – review and editing (supporting). **Tetsuji Yamaguchi:** data curation (equal), writing – review and editing (supporting). **Yoshiyuki Takei:** investigation (supporting), writing – review and editing (supporting). **Motoh Iwasa:** formal analysis (equal), investigation (equal), writing – review and editing (supporting). **Takefumi Yamashita:** data curation (lead), writing – review and editing (supporting). **Masatoshi Watanabe:** data curation (equal), formal analysis (equal), methodology (equal), writing – review and editing (supporting). **Hayato Nakagawa:** investigation (supporting), writing – review and editing (supporting). **Noriko Yasuhara:** data curation (equal), writing – review and editing (equal).

## Funding

This study was supported by KAKEN, 22K07982 and 25K11201 to JT and AE.

## Ethics Statement

The study protocol was approved by the ethics committee of Mie University Hospital and Omiya City Clinic (H2023‐090).

## Consent

Written informed consent was obtained from all subjects at the time of blood sampling.

## Conflicts of Interest

The authors declare no conflicts of interest.

## Supporting information


**Figure S1:** Original membranes for WB, Figure 4B,F.

**Table S1:** Oligonucleotides for mRNA expression.

## Data Availability

Data cannot be made publicly available because it contains identifying information. These restrictions have been imposed by the Ethics Committee of Mie University. Data can be made available upon request from the Clinical Research Review Committee of Mie University Hospital (contact via kk-sien@med.mie-u.ac.jp) for researchers who meet the criteria for access to confidential data.
