# Supplementary material for: Plexin Domain Containing 2, a Protein Specifically Expressed and Elevated in Human Pancreatic Cancer Tissue and Serum, Influences Cell Proliferation by Correlating With Cortactin
Source: Cancer Med. 2025 Dec 9;14(23):e71459. doi: 10.1002/cam4.71459 (PMC12688484; doi:10.1002/cam4.71459)
Supplement: Supplementary file 2 — Table S1: Oligonucleotides for mRNA expression. [file CAM4-14-e71459-s002.docx]

**Supplementary Table 1. Oligonucleotides for mRNA expression**

| PLXDC2 | forward | TTCTCAAGGCGGTAGACACGA |
| --- | --- | --- |
|  | reverse | CGATCTGAGTGTTATTGTCCTGC |
| Cortactin | forward | GTGGTTTTGGCGGCAAGTATG |
|  | reverse | CTCTCTGTGACTCGTGCTTCT |
| MYC | forward | GGCTCCTGGCAAAAGGTCA |
|  | reverse | CTGCGTAGTTGTGCTGATGT |
| POU5F1 | forward | CTTGAATCCCGAATGGAAAGGG |
|  | reverse | GTGTATATCCCAGGGTGATCCTC |
| MKI67 | forward | GAAAGAGTGGCAACCTGCCTTC |
|  | reverse | GCACCAAGTTTTACTACATCTGCC |
| β2 microglobulin | forward | CGAGACCGATGTATATGCTTGC |
|  | reverse | GTCCAGATGATTCAGAGCTCCA |
